# MdMAPKKK1 Regulates Apple Resistance to *Botryosphaeria dothidea* by Interacting with MdBSK1

**DOI:** 10.3390/ijms23084415

**Published:** 2022-04-16

**Authors:** Nan Wang, Yingshuang Liu, Chaohua Dong, Yugang Zhang, Suhua Bai

**Affiliations:** 1 Key Laboratory of Plant Biotechnology of Shandong Province, College of Life Sciences, Qingdao Agricultural University, Qingdao 266109, China; wangnan817@stu.qau.edu.cn (N.W.); dongchaohua@qau.edu.cn (C.D.); 2 College of Horticulture, Qingdao Agricultural University, Qingdao 266109, China; liuyingshuang@stu.qau.edu.cn (Y.L.); zyg4458@163.com (Y.Z.); 3 Shandong Province Key Laboratory of Applied Mycology, Qing Agricultural University, Qingdao 266109, China

**Keywords:** apple disease, disease resistance, *Malus domestica*, MAPKKK, plant immunity, ring rot

## Abstract

Plant MAPK cascade performs a critical role in the regulation of plant immunity and disease resistance. Although the function of MAPK cascade in immunity regulation is partially conserved between different species, the mechanism varies in different host and pathogen combinations. To date, the MAPK cascade function of woody plants in the regulation of disease resistance has seldom been reported. Here, we present evidence to show that apple *MdMAPKKK1* performed an important role in the regulation of apple resistance to *Botryosphaeria dothidea*, the causal agent of apple ring rot. *B. dothidea* infection leads to enhanced *MdMAPKKK1* expression and MAPK cascade activation, indicating that the MAPK cascade is involved in the defense against *B. dothidea*. *MdMAPKKK1* overexpression-induced pathogen-independent cell death. *MdMAPKKK1* silencing decreases the resistance of apple calli and fruits to *B. dothidea*. Further analysis indicates that MdMAPKKK1 can bind MdBSK1 and is likely phosphorylated by it. The MdBSK1-mediated phosphorylation of *MdMAPKKK1* is important for resistance to *B. dothidea*. These results collectively indicate that apple resistance to *B. dothidea* is regulated by the interaction between MAPKKK1 and MdBSK1.

## 1. Introduction

Apple ring rot is one of the main diseases of apple, which seriously damages apple production in China. Fungus *Botryosphaeria dothidea* is the causal agent of ring rot, which is a hemibiotrophic pathogen. It causes local proliferation and wart formation on the epidermis of branches and stem. In serious cases, *B. dothidea* leads to the death of infected shoots and stems. *B. dothidea* also infects the apple fruits, resulting in a ring necrosis spot around the infection site [1]. Although some genes associated with apple defense against *B. dothidea* have been identified [2,3,4,5,6], the detailed mechanism underlying disease resistance remains to be elucidated.

In recent years, some studies have reported that several transcription factors were involved in apple resistance to *B. dothidea*. Apple transcriptional factor MdMYB73, for example, interacts with MdWAKY31 and confers increased resistance to *B. dothidea* in apples via the salicylic acid pathway [7]. MdERF11 is another apple nucleus-localized ERF transcription factor [8]. Silencing the *MdERF11* gene in apple calli resulted in reduced resistance to *B. dothidea*, whereas ectopic expression of *MdERF11* in Arabidopsis also exhibited enhanced resistance to *B. dothidea*. Further analysis indicated that the effect of MdERF11 on plant resistance to *B. dothidea* is attributed to its role in regulating the SA synthesis pathway [8]. MdPUB29 is an apple U-box E3 ligase which is a positive regulator of apple resistance to *B. dothidea* by regulating SA synthesis and signaling [4]. It can be ubiquitinated and degraded by E3 Ligase MdPOB1, leading to reduced resistance to *B. dothidea* [4]. Although these studies reported several genes regulating the SA synthesis or SA signaling pathway, little is known about the upstream molecules recognizing pathogens and relaying the infection signal.

Mitogen-activated protein kinase (MAPK) cascade performs vital roles in regulating plant immunity, which is responsible for receiving pathogen infection signals from upstream molecules, then amplifying and transmitting the signals to downstream components through a series of phosphorylation events. MAPK cascade is also one of the key targets attacked by pathogens in plants [9]. MAPK cascade includes three groups of protein kinases, MAPK, MAPK kinase (MAPKK or MEK), and MAPK kinase kinase (MAPKKK). MAPK is activated by MAPKK (or MEK), while the latter is activated by upstream MAPKKK. The three highly conservative kinases extend the upstream signal step by step and then transmit them to the nucleus, leading to the occurrence of immune responses, such as hypersensitive response (HR), expression of defense genes, cell wall strengthening, and phytohormone synthesis. The immune responses ultimately determine plant resistance to diseases. At present, two MAPK pathways involved in plant immunity have been identified. The first one is composed of MAPKKK1, MAPKK1/MAPKK2, and MAPK4, which negatively regulates plant immunity [10,11]. The second cascade consists of MAPKKK5/MAPKKK3, MAPKK4/MAPKK5, and MAPK3/MAPK6 [12,13,14]. The second cascade reaction is widely involved in the regulation of plant disease resistance by activating defense gene expression, phytoalexin, ethylene biosynthesis, and stomatal immunity [15,16,17]. MAPKKK is the first kinase of MAPK cascade which receives pathogen signals perceived by pattern recognition receptors (PRRs) and leads to activation of MAPK cascade through a series of phosphorylation events. Plant MAPKKKs are highly divergent and can be divided into two large subfamilies: MAPKKK-like kinases and Raf-like kinases [18]. In Arabidopsis, MAPKKK5 interacts with and is phosphorylated by its upstream RLCK family protein PBL27 [19]. Phosphorylation of MAPKKK5 by PBL27 is essential for chitin-induced MAPK activation in plants [19]. However, subsequent studies reported that chitin-induced MAPKKK5 activation was mediated by PBL19 [13], whereas flg22-induced MAPKKK5 activation depends on the interaction between BSK1 and MAPKKK5 [14]. A similar MAPK cascade was also found in rice. OsMAPKK18 is an ortholog of Arabidopsis MAPKKK5 in rice. OsRLCK185, an ortholog of Arabidopsis PBL27 is phosphorylated by OsCERK1 and phosphorylates OsMAPKKK18 and OsMAPKKK11, then OsMAPKK18 phosphorylated OsMAPKK4, leading to activation of downstream OsMAPK3 and OsMAPK6 and downstream immune responses [20]. MAPK cascade has been reported to be involved in the regulation of apple disease resistance by modulating the SA signaling pathway [21]. MdMAPKK4-MdMAPK3 negatively regulates apple resistance to the Glomerella leaf spot (GLS) by phosphorylating MdWRKY17, a transcriptional factor. Phosphorylated MdWRKY17 promotes the expression of MdDMR6 which subsequently degrades SA and leads to reduced resistance to GLS [21]. However, to date, complete MAPK cascade regulating apple immunity is not established. Although apple MAPKKKs have been identified and analyzed throughout the whole genome [22], whether MAPK cascade regulates apple resistance to *B. dothidea* is not known. 

In the present study, we performed homologous BLAST against the apple genome database to search the apple *MAPKKK* genes involved in the plant immunity and analyzed their expression responses to *B. dothidea* infection. An apple *MAPKKK* gene (designated as *MdMAPKKK1*) was found to be up-regulated by *B. dothidea*. Further analysis indicates that MdMAPKKK1 interacts with BSK1 and modulates the resistance to *B. dothidea*.

## 2. Results

### 2.1. Apple MAPK Cascade Is Involved in the Defense against B. dothidea 

**Figure 1:**
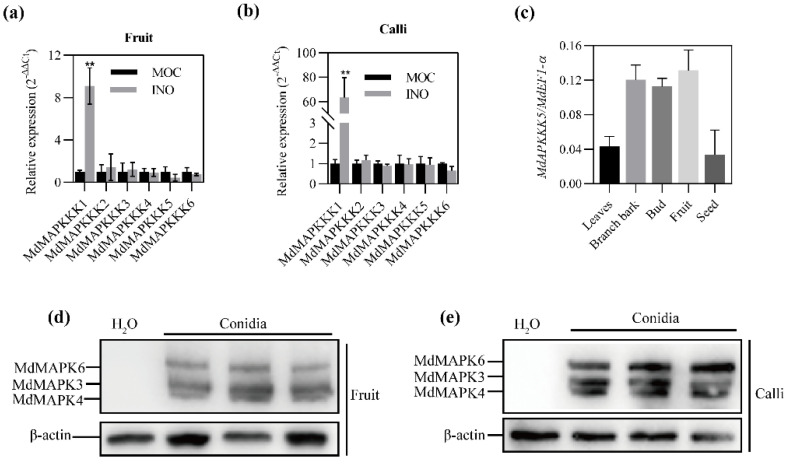
*MdMAPKKKs’* expression and the activation of the apple MAPK cascade. (**a**) and (**b**) relative expression of *MdMAPKKKs* in the apple fruits and calli inoculated with *B. dothidea*. MOC, the apple fruits 130 days after full blossom (DAFB) or 3-week-old calli mock-inoculated with sterilized double distilled water. INO, the apple fruits or 3-week-old calli inoculated with *B. dothidea*. The inoculated or mock-inoculated calli and fruit tissues around the inoculation sites were collected 48 h after inoculation for gene expression analysis and immunoblotting. The mock-inoculated fruits and calli were used as control. The data were analyzed using two-way ANOVA followed by Sidak’s test. **, *p* < 0.01. (**c**) Relative expression of the *MdMAPKKK1* gene in different apple tissues. (**d**) and (**e**) the MAPK cascade activation in fruits and calli inoculated with *B. dothidea* as revealed by immunoblotting with anti-PERK. MdMAPK3, MdMAPK4, and MdMAPK6 refer to putative apple homologues of AtMAPK3, AtMAPK4, and AtMAPK6, respectively.

To examine if the apple MAPK cascade is responsive to *B. dothidea* infection, we identified apple homologues of the MAPKKK protein that is the first component of MAPK cascade and is associated with plant resistance. We searched against the apple genome and cloned six *MAPKKK* genes for expression analysis (designated as *MdMAPKKK1-MdMAPKKK6*). Of the six genes, *MdMAPKKK1* (MD02G1167500) is homologous to *AtMAPKKK5* which is involved in bacterial and fungal immunity in Arabidopsis [13,14]. *MdMAPKKK2* (MD04G1217700) and *MdMAPKKK3* (MD12G1234900) are the homologues of *EDR1* that encodes a Raf-like MAPK kinase kinase (MAPKKK) and functions as a negative regulator of plant defense [18,23]. *MdMAPKKK4* (MD12G1219600), *MdMAPKKK5* (MD11G1055100), and *MdMAPKKK6* (MD03G1053500) encode the homologue of the *NPK1-related protein kinase 1* gene (*ANP1*) that is required for elicitor-induced oxidative burst and immunity [24,25] (Supplementary Figure S1). Among these *MAPKKK* genes, *MdMAPKKK1* is the only one that demonstrated the enhanced expression in apple fruits and the ‘Orin’ calli 48 h after inoculation with *B. dothidea*, suggesting the involvement of *MdMAPKKK1* in the apple resistance to *B. dothidea* (Figure 1a,b). A tissue expression analysis showed that *MdMAPKKK1* was expressed in all examined tissues with different abundance (Figure 1c). To further determine the involvement of MAPK cascade in the defense against *B. dothidea*, we analyzed the activation of MAPK cascade in fruits inoculated with *B. dothidea*. The activation of MAPK cascade was observed 48 h after *B. dothidea* inoculation (Figure 1d). The mock-infected control did not exhibit the MAPK activation (Figure 1d,e), indicating the activation was not a result of mechanical stimuli. The MAPK activation was also observed in inoculated apple calli (Figure 1e). The results indicate that MAPK cascade is responsive to the infection of *B. dothidea*, and *MdMAPKKK1* may be important for defense responses to *B. dothidea*.

### 2.2. MdMAPKKK1 Overexpression Leads to Death of Plant Cell

**Figure 2:**
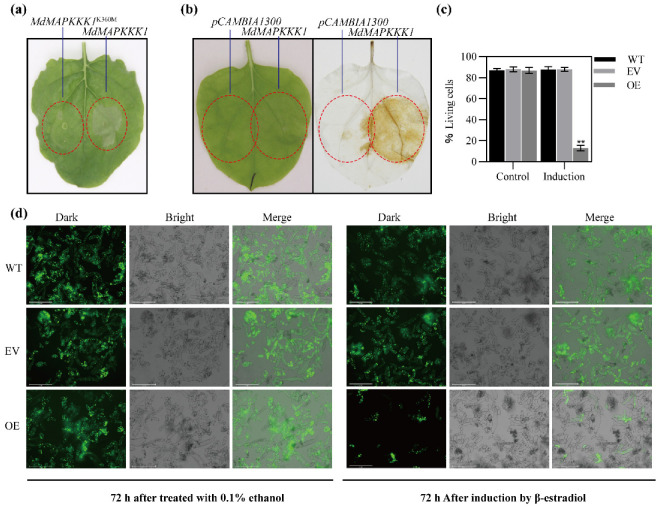
*MdMAPKKK1* overexpression caused cell death. (**a**) *Nicotiana* leaves infiltrated with *Agrobacterium* harboring pCAMBIA1300-*MdMAPKKK1* exhibited tissue necrosis 48 h after infiltration, but those infiltrated with *Agrobacterium* harboring pCAMBIA1300-*MdMAPKKK1^K360M^* did not. (**b**) *MdMAPKKK1* overexpression led to H_2_O_2_ accumulation. Left: *Nicotiana* leaves 24 h after infiltration with GV3101 containing pCAMBIA1300 or pCAMBIA1300-*MdMAPKKK1* plasmids; right: *Nicotiana* leaves were stained with 3, 3′-diaminobenzidine (DAB) 24 h after infiltration. (**c**) The % live cells after induction of *MdMAPKKK1* expression by β-estradiol. Control: the calli treated with 0.1% ethanol (β-estradiol solvent). Induction: the calli treated with 5 μM β-estradiol. **, *p* < 0.01. (**d**) *MdMAPKKK1* expression-induced apple cell death. Live cells were stained with fluorescein diacetate (FDA). WT: wild type apple cell from ‘Orin’ calli; EV: apple cells transformed with pER8 vector; OE: apple cells transformed with pER8-*MdMAPKKK1*. *MdMAPKKK1* expression was induced by 5 μM β-estradiol. The scale bars represent 275 nm.

Previous research indicated that heterologous overexpression of *AtMAPKKK5* induced cell death of *Nicotiana benthamiana* leaves [14,19]. To test if *MdMAPKKK1* also induces cell death, *MdMAPKKK1* was transiently overexpressed in *Nicotiana* leaves. Tissue necrosis was observed 2 d after infiltration with *Agrobacterium* harboring the *MdMAPKKK1* gene under the control of the 35S promoter (Figure 2a), which is comparable to the tissue necrosis induced by *AtMAPKKK5* [14,19]. The necrosis area was accompanied with ROS accumulation (Figure 2b). We mutated the putative ATP binding site of the MdMAPKKK1 protein from Lys360 to Met360 (K360M). The mutated *MdMAPKKK1* (*MdMAPKKK1^K360M^*) did not induce cell death (Figure 2a). To determine if *MdMAPKKK1* overexpression can also induced cell death in native organism, cDNA of *MdMAPKKK1* was inserted into inducible pER8 vector and introduced into apple calli. After antibiotic screening and PCR confirmation, positive calli were treated with β-estradiol to induce *MdMAPKKK1* expression. Obvious cell death was observed 72 h after induction by β-estradiol (Figure 2c,d), suggesting a relative lower basal expression of the *MdMAPKKK1* gene during cell normal growth under the non-infection condition.

### 2.3. MdMAPKKK1 Silencing Impairs Apple Resistance to B. dothidea

**Figure 3:**
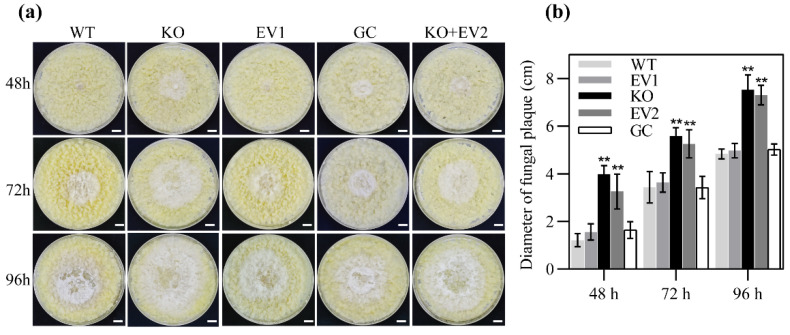
*B. dothidea* growth on calli. (**a**) Apple calli inoculated with *B. dothidea*. The 2-week-old calli were used for inoculation. The inoculation was performed by placing the mycelial discs on the center of the calli. The scale bars represent 9.0 mm. WT: wild type; KO: mutated calli generated by CAS9/CRIPR system. EV1: empty vector for gene editing; GC: genetic complemented calli. KO+EV2: KO calli transformed with empty vector for genetic complement. (**b**) Fungal growth evaluated by measuring the diameter of fungal plates. The bars represent means (± SD) from five calli dishes (*n* = 5). **, *p* < 0.01.

To determine whether *MdMAPKKK1* is involved in the defense of apple against *B. dothidea*, we knocked out the *MdMAPKKK1* gene in apple callus using the CRISPR/Cas9 system (KO) with the empty CRISPR/Cas9 vector as a control (EV) (Supplementary Figure S2a–c). The *MdMAPKKK1* gene was successfully mutated in KO calli (Supplementary Figure S2b,c). We examined the resistance of KO and EV calli to *B. dothidea*. The KO clones of apple callus used for infection analysis have at least two different mutations in CDS of *MdMAPKKK1* and no WT sequence of the *MdMAPKKK1* gene was detected. Target sites of the *MdMAPKKK1* gene in the KO clones exhibited deletion or insertion of bases, leading to interruption of CDS (Supplementary Figure S2b,c). No significantly different phenotype was observed in KO, EV, and WT calli. The 2-week-old calli of KO, EV, and WT were inoculated with *B. dothidea*. The diameters of radial mycelial extension were measured as an index to evaluate the resistance of calli. The fungal growth exhibited a significant difference between KO and EV or between KO and WT (Figure 3a,b). A significant larger mycelial diameter and faster mycelial growth were observed in KO calli compared with that in EV and WT calli (Figure 3a,b). To further confirm that the reduced resistance resulted from the *MdMAPKKK1* knockout, we changed the PAM site using site-directed mutagenesis technology (Supplementary Figure S2d) and constructed a new vector to express the *MdMAPKKK1* gene under control of its native promoter. Then, the new construct was introduced into KO calli. The expression of *MdMAPKKK1* completely recovered the resistance of KO calli to *B. dothidea* (Figure 3a,b).

**Figure 4:**
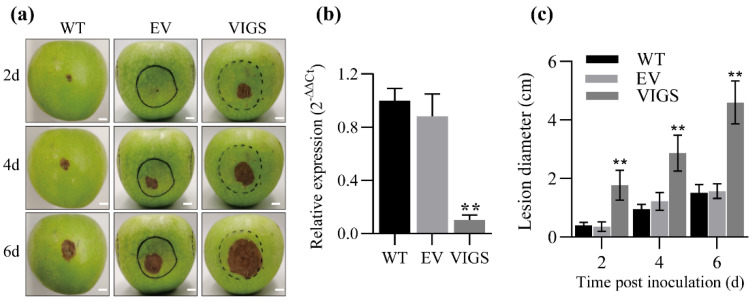
The effects of *MdMAPKKK1* on apple fruit resistance to *B. dothidea*. (**a**) Apple fruits inoculated with *B. dothidea*. The fruits were collected from ‘Granny Smith’ cultivar 130 DAFB. Inoculation was performed at 7 d after infiltration. The circles and broken cycles represent the area that the *Agrobacterium* suspension initially spread. The lesions were evaluated at 2 d, 4 d, and 6 d after inoculation with *B. dothidea*. WT: wild type; EV: pTRV1+pTRV2; VIGS: pTRV1+pTRV2-*MdMAPKKK1*. Scale bars represent 10.0 mm. (**b**) Relative expression of *MdMAPKKK1* normalized to *MdEF1-α* in fruits 7 d after agro-infiltration. The bars represent means ± SD (*n* = 3). ** *p* < 0.01. (**c**) Lesion diameter of fruit after inoculation. The bars represent means ± SD (*n* = 5). The data were subjected to two-way ANOVA analysis followed by Sidak’s test. Double asterisks represent significant difference compared with WT (** *p* < 0.01).

To further determine the effect of *MdMAPKKK1* on the resistance to *B. dothidea*, we performed VIGS to knock down the *MdMAPKKK1* gene in fruits. Then, the fruits were inoculated with *B. dothidea* and lesions were evaluated at 48, 96 and 144 hpi. *MdMAPKKK1* expression was significantly reduced after VIGS (Figure 4a,b). Reduction in *MdMAPKKK1* expression led to increased lesion of fruits by *B. dothidea* infection (Figure 3a,c), indicating that *MdMAPKKK1* positively regulated apple resistance to *B. dothidea*.

### 2.4. MdMAPKKK1 Interacts with BSK1-1 and BSK1-2

**Figure 5:**
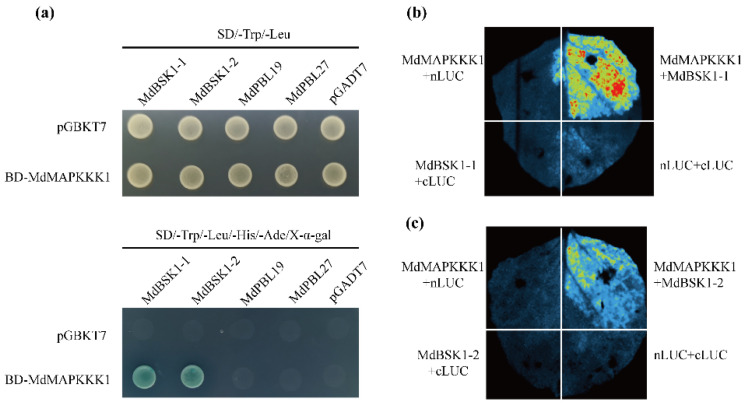
Interaction between BSK1 and MdMAPKKK1. (**a**) Point-to-point verification of the interaction between MdMAPKKK1 and MdBSK1 by yeast two-hybrid. The constructs were transformed into yeast Y2HGold stain and 10 μL suspension of yeast containing each pair of constructs dotted on the indicated SD medium. (**b**) and (**c**) split luciferase assay of the interaction between BSK1 and MdMAKKK1. *N. benthamiana* leaf co-infiltrated with *Agrobacterium* containing CLuc-MdBSK1 and MdMAKKK1K360M-NLuc. The luminescence images were captured using Newton 7.0 system.

To explore the proteins interacting with *MdMAPKKK1*, we performed a yeast two-hybrid screen against the apple cDNA library. After excluding the effect of autoactivation, we obtained 104 positive clones. Of these clones, 24 proteins have been reported to be associated with plant immunity, including two BSK proteins (designated as MdBSK1-1 and MdBSK1-2). MdBSK1-1 and MdBSK1-2 share high amino acid and nucleotide sequence identity (Supplementary Figure S3). Subsequently, we performed point-to-point verification and a split luciferase assay. Both of the two experiments confirmed the interaction between MdMAPKKK1 and MdBSKs (Figure 5a–c). LUC fluorescence was observed in the *Nicotiana* leaves co-expressing *MdMAPKKK1* and *MdBSK1-1* or co-expressing *MdMAPKKK1* and *MdBSK1-2*, indicating that MdBSK1-1 and MdBSK1-2 are the genuine proteins interacting with MdMAPKKK1. 

### 2.5. BSK1 Phosphorylates MdMAPKKK1

**Figure 6:**
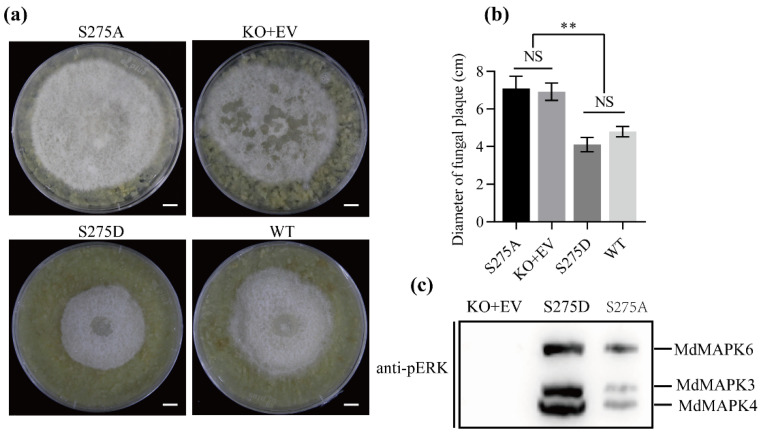
MdMAPKKK1 phosphorylation by MdBSK1 affected the resistance of apple callus to *B. dothidea*. (**a**) The putative phosphorylation site of MdMAPKKK1 targeted by MdBSK1 affects the resistance of apple callus to *B. dothidea*. The 2-week-old week calli were inoculated with *B. dothidea*. The photos were taken at 5 d after inoculation. Scale bars represent 8.0 mm. S275A: *MdMAPKKK1*-KO callus was transformed with *MdMAPPPK1^S275A^* and inoculated with *B. dothidea*; KO+EV: *MdMAPKKK1*-KO callus was transformed with pRI101 empty vector; S275D: *MdMAPKKK1*-KO callus was transformed with *MdMAPPPK1^S275D^*; WT: wild type callus. (**b**) Quantitation of calli resistance to *B. dothidea* by measuring the diameter of fungal plaque. NS: no significant difference. ** *p* < 0.01. (**c**) The MdMAPKs activation in the calli inoculated with *B. dothidea* as revealed by immunoblotting with anti-PERK.

The interaction between MdMAPKKK1 and MdBSK1 leads to a speculation that MdBSK1 phosphorylates MdMAPKKK1. We aligned the amino acid sequence of MdMAPKKK1 with Arabidopsis MAPKKK5 protein. We found that MdMAPKKK1 has a putative phosphorylation site at Ser275 corresponding to Ser289 of Arabidopsis MAPKKK5 which was reported to be the BSK1 target site [14]. However, the MdMAPKKK1 protein has no phosphorylation sites corresponding to Ser599 and Ser682 of Arabidopsis MAPKKK5 which are critical for MAPKKK5 function [13] (Supplementary Figure S4). To determine if mutation of the putative phosphorylation site targeted by MdBSK1 influences the resistance to *B. dothidea*, we introduced *MdMAPKKK1^S275A^* and *MdMAPKKK1^S275D^* into the *MdMAPKKK1*-KO calli. *MdMAPKKK1^S275A^* causes a loss of phosphorylation, while *MdMAPKKK1^S275D^* mimics constitutive phosphorylation [14]. The expression of *MdMAPKKK1^S275D^* under the control of the native promoter recovered the resistance to *B. dothidea* of MdMAPKKK1-KO calli. However, the expression of *MdMAPKKK1^S275A^* did not affect the resistance of calli to *B. dothidea* (Figure 6a,b). Next, we examined the activation of MAPKs after inoculation with *B. dothidea*. We found that *B. dothidea* induced MAPKs activation in both *MdMAPKKK1^S275D^*- and *MdMAPKKK1^S275A^*-expressed calli, but MAPK activation in *MdMAPKKK1^S275A^*-expressed callus was significantly reduced compared with that in *MdMAPKKK1^S275D^*-expressed callus. The results indicate that MdBSK1 mediates the signal transduction of *B. dothidea* infection to MdMAPKKK1 by phosphorylation (Figure 6c).

### 2.6. BSK1 Silencing Decreases Apple Resistance to B. dothidea

**Figure 7:**
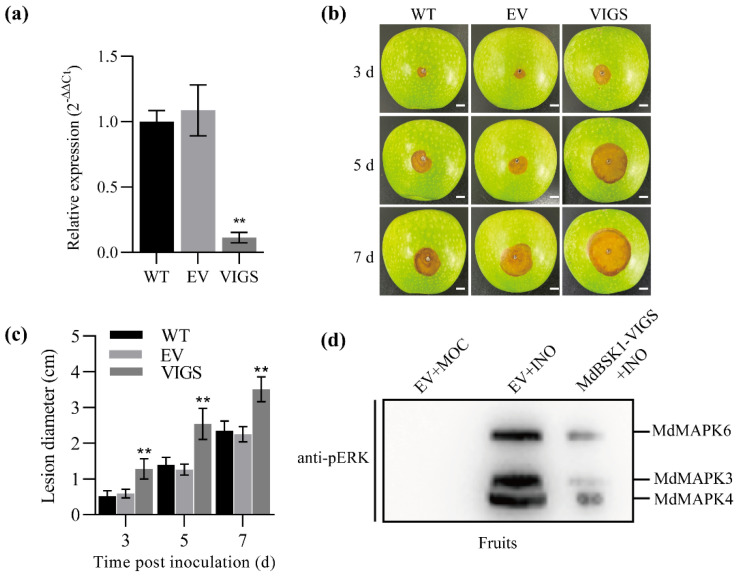
The effect of *MdBSK1* on apple immunity. (**a**) *MdBSK1* silencing reduced apple resistance to *B. dothidea*. Scale bars represent 10.0 mm. WT: the apple fruits were not treated before inoculation with *B. dothidea*. EV: the fruits were infiltrated with a mixture of *Agrobacterium* harboring pTRV1 and pTRV2, and followed by inoculation with *B. dothidea*. VIGS: the fruits were infiltrated with a mixture of *Agrobacterium* harboring pTRV1 and pTRV2-*MdBSK1*, and followed by inoculation with *B. dothidea*. (**b**) Relative expression of *MdBSK1* in VIGS-fruits and controls. The samples were collected 7 d after infiltration. The tissues from three fruits were mixed equally as a sample for RNA extraction and cDNA synthesis. Three biological replicates were set up for qRT-PCR assay (*n* = 3). The bars represent means ± SD (*n* = 3), ** *p* < 0.01. (**c**) Quantitation of resistance to *B. dothidea* by measuring lesion diameter of fruit after inoculation. ** *p* < 0.01. (**d**) The MAPK cascade activation in fruits inoculated with *B. dothidea* as revealed by immunoblotting with anti-PERK.

To address if *MdBSK1* influences apple resistance to *B. dothidea*, we silenced the *MdBSK1* gene using VIGS. We constructed pTV2 containing 300 bp fragment of MdBSK1 for the VIGS experiment. *MdBSK1* expression can be effectively knocked down by VIGS (Figure 7a). We inoculated *MdBSK1*-silenced fruits with *B. dothidea* and observed significantly decreased fruits resistance to *B. dothidea* (Figure 7b,c), indicating that *MdBSK1* contributed to apple resistance of *B. dothidea*. To further determine the effect of *MdBSK1* on activation of MAPK cascade, we detected the MAPKs activation in the *MdBSK1*-silenced apple fruits. MAPK activation was significantly reduced after inoculation (Figure 7d). 

## 3. Discussion

The *MAPKKK* genes have been reported to be involved in the immune defense in model plants such as Arabidopsis and *Nicotiana* plant. However, if *MAPKKKs* perform similar roles in apple plant immunity remains unclear. Here, we report that *MdMAPKKK1* functions as a positive regulatory factor on apple resistance to *B. dothidea*, and exhibits a function pattern different from its homologous genes in model plants. The results presented here provide new insight into MAPKKK-mediated apple defense mechanisms against *B. dothidea*.

The *MdMAPKKK1* gene can be induced by *B. dothidea* in apple fruit and calli, indicating that *MdMAPKKK1* is involved in the apple defense responses against *B. dothidea*. The increased expression of *MdMAPKKK1* may be responsible for MAPK activation and contribute to the defense against pathogens. The induction of *MAPKKK* genes by pathogens was also observed in Arabidopsis *MAPKKK5* which was induced by *Golovinomyces cichoracearum* [14] and *Pseudomonas syringae pv. tomato* DC3000 [13], and an increase in *MAPKKK5* expression leads to enhanced MAPK activation [13]; which suggests that MAPKKK protein induction by pathogens is a conserved mechanism in plant immune defense.

MdMAPKKK1 is highly homologous to MAPKKKα of *Nicotiana* plants and Arabidopsis MAPKKK5 which have been reported to be involved in the resistance to bacterial and fungal pathogens, including biotrophic, hemibiotrophic, and necrotrophic pathogens, such as *P. syringae pv. tabaci* (hemibiotroph) [26], *P. syringae pv. tomato* (hemibiotroph) [14], fungal pathogen *G. cichoracearum* (biotroph) [14], *B. cinerea* (necrotroph) [13], and *Alternaria brassicicola* (necrotroph) [19]. Arabidopsis MAPKKK5 phosphorylates and activates MAPKK4/MAPKK5. The phosphorylated MAPKK4/MAPKK5 further activates MAPK3 and MAPK6. Then, MAPK3/MAPK6 further activates the defense gene expression, phytoalexin and ethylene biosynthesis, and stomatal immunity [13,15,16,17]. As for apple plant, only MdMAPKK4 and MdMAPK3 were reported to be involved in the disease resistance regulation [21]. Moreover, different from model plants, apple MdMAPKK4/MdMAPK3 negatively modulates GLS resistance. Apple MdMAPKK4 phosphorylated MdMAPK3 which in turn phosphorylated WRKY17. The phosphorylated WRKY17 binds to the promoter of the *MdDMR6* gene and promotes *MdDMR6* expression. MdDMR6 leads to degradation of salicylic acid (SA) and decreased disease resistance [21]. However, our results demonstrate the positive regulation of *MdMAPKKK1* on apple disease resistance to *B. dothidea.* Upon *B. dothidea* infection, MdBSK1 phosphorylated MdMAPKKK1, leading to MdMAPKs activation and positively modulated apple resistance to *B. dothidea*. These results indicate that apple MAPK cascade differentially regulated the resistance to different fungal pathogens.

Cell death appears in both susceptible and resistant plants subjected to pathogen attack. In susceptible plants, pathogen infection results in cell death that develops into disease symptoms. In resistant plants, the host resistance (R) protein recognizes the pathogen avirulence (Avr) protein leading to host hypersensitive responses (HR) in a form of localized programmed cell death (PCD). PCD limits proliferation of biotrophic pathogens, but is conducive to facultative necrotrophic pathogens [26,27]. *MdMAPKKK1* overexpression results in cell death independent of pathogens cell death, indicating that *MdMAPKKK1* overexpression leads to autoimmunity. A similar phenomenon was also observed in *NbMAPKKKα*, *MAPKKKγ*, *MAPKKKβ*, *SlMAPKKKα*, and *AtMAPKKK5* [13,14,26,28]. Different from these MAPKKKs, Arabidopsis *MAPKKK1* negatively regulated Arabidopsis immunity and cell death. Arabidopsis *mapkkk1* mutant shows seedling lethality [10,29]. However, HR cell death is not always essential or sufficient for disease resistance. Whether *MdMAPKKK1* overexpression-induced cell death is associated with apple resistance to *B. dothidea* remains to be known. Although previous studies have demonstrated that *MAPKKKα* and *MAPKKK5* are also involved in PCD, their roles in regulation on disease resistance are different. *MAPKKKα* negatively regulated the resistance of *Nicotiana* plant to virulent pathogen *P. syringae pv. tabaci* but contributed to the resistance of *Nicotiana* plant to avirulent *P. syringae pv. tabaci* (avrPto) [26]. In Arabidopsis, *MAPKKK5* positively modulated Arabidopsis resistance to virulent and avirulent strains of the bacterial pathogen *P. syringae pv. tomato* DC3000, and the fungal pathogen *G. cichoracearum* and *Botrytis cinerea* [13,14]. These reports indicate that the contribution of MAPKKKs induced-cell death to the disease resistance depends on different pathogens. *B. dothidea* is a hemibiotroph fungus, which means that *MdMAPKKK1*-induced cell death is beneficial for limiting the proliferation of *B. dothidea* in the early biotrophic phase. This contributes to disease resistance. However, once the pathogenic fungus *B. dothdea* breaks the initial line of immune defense in the later necrotrophic stage the fungus utilizes the dead cells for nutrient acquisition, which is beneficial to the growth of pathogenic fungus. 

In Arabidopsis, it has been reported that MAPKKK5/MAPKKK3 receives the signal mediated by receptor-like cytoplasmic kinases RLCK [13,14,19]. Pathogen associated molecular patterns (PAMPs) such as chitin, flg22, and elf18 are recognized by pattern recognition receptor (PRR) and trigger the MAPK cascade activation [12]. However, the signal component linking the PRR and MAPK cascade in different studies are different. BSK1, PBL27 and PBL19 were reported to be the molecules linking PRR and MAPKKK5 in different studies [13,14,19], respectively. Here, we found apple MAPKKK1 interacts with BSK1 homologous protein MdBSK1, but did not interact with the apple homologous proteins of Arabidopsis PBL27 and PBL19, MdPBL27 and MdPBL19. The results suggest that apple MAPK cascade functions differentially from that of Arabidopsis.

Taken together, MdMAPKKK1 exhibited a positive regulation on the apple resistance to *B. dothidea* and this regulation depends on MdBSK1. MdBSK1 mediated the transduction of pathogen signal by phosphorylating MdMAPKKK1. Both MdMAPKKK1 and MdBSK1 contribute to apple resistance to *B. dothidea*.

## 4. Materials and Methods

### 4.1. Plant Materials and Pathogen

Apple fruits (‘Granny Smith’ cultivar) were obtained from Laixi elite seed-breeding farm, Qingdao, China. ‘Orin’ calli were cultured in Y1 medium (MS 4.4 g L^−1^, sucrose 30 g l^−1^, 6-BA 0.225 mg L^−1^, 2,4-D 1 mg L^−1^ pH 5.8). *B. dothidea* was cultured on the PDA medium at 25 °C. To obtain conidia, the fresh mycelia were inoculated on young apple fruits 50 DAFB and kept at room temperature for 10 days. The conidia were washed off with sterilized distilled water and adjusted to a concentration of 1.0 × 10^7^ conidia ml^−1^. 

### 4.2. Quantitative Reverse Transcription-PCR and MAPK Activation Assay

Prior to inoculation, the apple fruits collected at 130 DAFB were surface-sterilized with 75% ethanol and stabbed with a needle. The fruits were inoculated with 10 μL conidia of *B. dothidea* (1 × 10^7^ conidia mL^−1^) and maintained at 25 °C. The peel and flesh around inoculation sites were collected at 48 h after inoculation and used for total RNA and protein extraction. The ‘Orin’ calli were inoculated by spraying conidia of *B. dothidea* (1 × 10^7^ conidia mL^−1^) in petri dishes of 9 cm in diameter. The calli were collected 48 h after inoculation for RNA and protein extraction. A similar operation was performed with the sterilized double distilled water substituting for conidia and used as mock-inoculation. For tissue distribution of the *MdMAPKKK1* gene, leaves, the branch barks, buds, fruits, and seeds were collected from apple cultivar ‘Granny Smith’. The total RNA was extracted using the EASYspin plant RNA rapid extraction kit (Yuanpinghao, China) from the apple fruits and calli. First-strand cDNA were synthesized using the HiScript III 1st Strand cDNA Synthesis Kit (Vazyme, China) according to the manual provided by the manufacturer. A quantitative PCR (qPCR) was conducted on QuantStudio™ 5 Real-Time PCR System using ChamQ SYBR Color qPCR Master Mix (Vazyme, China). The RNA from three fruits or calli was equally mixed as one sample. The qPCR was performed with four biological replicates and each biological replicate included three technological repeats. The primers used for the qPCR are listed in Supplementary Table S2. The housekeeping gene MdEF1-α was used for normalization of target gene expression.

For the MAPK activation assay, fruit tissues or callus were ground in liquid nitrogen with a mortar and pestle, and then extracted with the protein extraction buffer (100 mM sodium acetate, pH 7.5, containing 100 mM NaCl, 1 mM EDTA, 1 mM phenylmethylsulfonyl fluoride, and 1% PVP). After centrifuge at 4 °C and 10,000× *g*, the supernatant containing 100 μg of total protein was resolved in a 12% SDS-PAGE gel and activated MAPKs were visualized by immunoblotting with anti-pERK monoclonal antibodies (Cell Signaling Technology, USA) as the first antibody at 1:2000 dilution. HRP-linked anti-rabbit IgG was used as secondary antibody at 1:5000 dilution. MAPKs were visualized by the BeyoECL Plus kit (Beyotime, China) and photographed using Newton Bio 7.0 (VILBER, France). 

### 4.3. Ectopic Transient Expression and Inducible Expression in ‘Orin’ 

*MdMAPKKK1* cDNA was inserted into pCAMBIA1300 to construct the expression vector. The resultant pCAMBIA1300-*MdMAPKKK1* was introduced into the *Agrobacterium* GV3101 strain. *Agrobacterium* containing pCAMBIA1300-*MdMAPKKK1* was infiltrated into *Nicotiana* leaves with a needleless syringe. Tissue necrosis was observed every day and stained with DAB 24 h after infiltration for examination of H_2_O_2_ accumulation. 

To overexpress *MdMAPKKK1* in ‘Orin’ calli, cDNA of the *MdMAPKKK1* CDS region was inserted into inducible vector pER8 and the resultant plasmid was transformed into the *Agrobacterium* EHA105 strain. *Agrobacterium* EHA105 containing pER8-*MdMAPKKK1* was suspended in induction buffer (MS 4.4 g L^−1^, sucrose 25 g L^−1^, acetosyringone 200 μM). Fresh calli were soaked into *Agrobacterium* harbouring-pER8-*MdMAPKKK1* for 10 min followed by culturing in Y1 medium for 3 d. Subsequently, the calli were transferred to Y2 medium (MS 4.4 g/L, sucrose 30 g L^−1^, 6-BA 0.225 mg L^−1^, 2,4-D 1 mg L^−1^, Hygromycin B 10 mg L^−1^, cefotaxime 200 mg L^−1^, Timentin 300 mg L^−1^) for selection. For inducible expression of *MdMAPKKK1*, the 2-week-old transformed calli were treated with 5 μM β-estradiol. The live cells were stained with FDA.

### 4.4. CRISPR/cas9 Mediated Gene Mutagenesis and Virus-Induced Gene Silencing

To silence the expression of the *MdMAPKKK1* gene in apple callus, a 20 bp target sequence of *MdMAPKKK1* was chosen using CCTOP online software and integrated into pHDE-35S-Cas9-kan vector to guide RNA generation. The resultant vector was introduced into the *Agrobacterium* EHA105 strain, and then transformed into apple callus following the procedure as described above. For mutation analysis, a DNA fragment of 500 bp around the target site was amplified using PCR with apple genomic DNA as a template, and sequenced. The inoculation was performed by placing the mycelial discs on the center of the callus and kept at 25 °C. The pathogen growth was monitored every day.

For *MdMAPKKK1* and *MdBSK1* silencing, a 300 bp cDNA fragment of the *MdMAPKKK1* and *MdBSK1* genes were inserted into pTRV2, respectively, and transformed into the *Agrobacterium* EHA105 stain. *Agrobacterium* containing pTRV2-*MdMAPKKK1* and pTRV1-harboring *Agrobacterium* were equally mixed and co-infiltrated into apple fruits 130 DAFB. The inoculation was performed at the infiltration site by injection with 10 μL conidia 7 d after infiltration. The resistance was evaluated by measuring the diameter of lesions.

### 4.5. Yeast Two Hybrid Assay

The cDNA fragment of *MdMAPKKK1* was inserted into pGBKT7. Then the pGBKT7-*MdMAPKKK1* and cDNA library of apple fused with pGADT7 were co-transformed into the Saccharomyces cerevisiae strain Y2HGold. The yeast cells were screened on SD/–Leu/–Trp and SD/–Ade/–His/–Leu/–Trp/X-α-gal medium. The positive clones were amplified using the vector primers and sequenced and subjected with point-to-point verification. Briefly, the full cDNA of the obtained targeted sequence was amplified and fused with pGADT7. The resultant plasmid and pGBKT7-*MdMAPKKK1* were co-transformed into Y2HGold and cultured on the SD/–Ade/–His/–Leu/–Trp/X-α-gal medium. The blue clones grown well were regarded as positive interaction in yeast cells.

### 4.6. Split Luciferase Assay

The split luciferase assay was performed according to the previously reported method (Zhou et al., 2018) with slight modifications. CDS of *MdBSK1* and *MdMAPKKK1* were inserted into pCAMBIA1300-cLUC, and pCAMBIA1300-nLUC, respectively. The constructs were transformed into the *Agrobacterium* GV3101 (pSOUP, p19) strain. The positive clones were cultured overnight (OD600 = 0.8). The bacteria were suspended with infiltration buffer (10 mM MgCl_2_, 10 mM MES, and 200 µM acetosyringone) and the OD600 was adjusted to 0.5. *Agrobacterium* containing pCAMBIA1300-cLUC-MdBSK1 and pCAMBIA1300-nLUC-*MdMAPKKK1* were equally mixed and co-infiltrated into *Nicotiana* leaves. Two days later, the leaves were detached and soaked in 0.5 mM D-luciferin for 10 min in the dark, and then were observed and photographed using the Newton 7.0 system.

## Data Availability

The data presented in this study are available in the article and the Supplementary Materials here.

## Supplementary Materials

The following supporting information can be downloaded at: https://www.mdpi.com/article/10.3390/ijms23084415/s1.
