# Supplementary material for: MdMAPKKK1 Regulates Apple Resistance to Botryosphaeria dothidea by Interacting with MdBSK1
Source: Int J Mol Sci. 2022 Apr 16;23(8):4415. doi: 10.3390/ijms23084415 (PMC9030329; doi:10.3390/ijms23084415)
Supplement: Supplementary file 1 [file ijms-23-04415-s001.zip › ijms-1679966-supplementary.pdf]

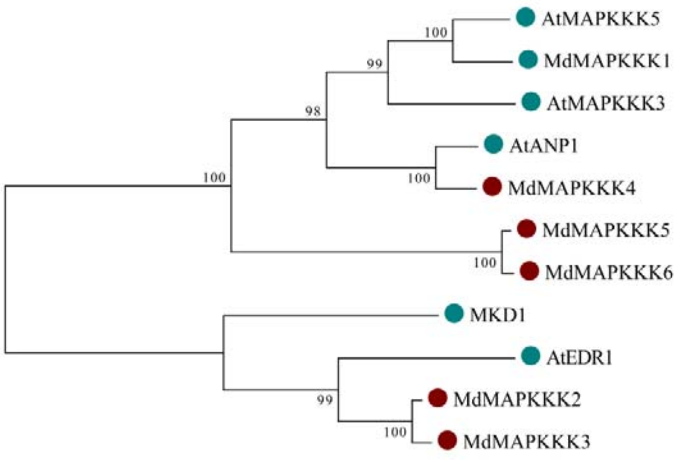

Figure S1. Phylogenetic tree constructed with MAPKKK proteins of apple and Arabidopsis. The accession numbers of the proteins used for phylogenetic tree were listed in supplementary Table S1. Phylogenetic tree were constructed using neighbor-joining method. The Bootstrap values for 1000 trials are indicated with numbers at the branch nodes. Blue dots indicate proteins from Arabidopsis and the dark red ones indicate proteins from apple.

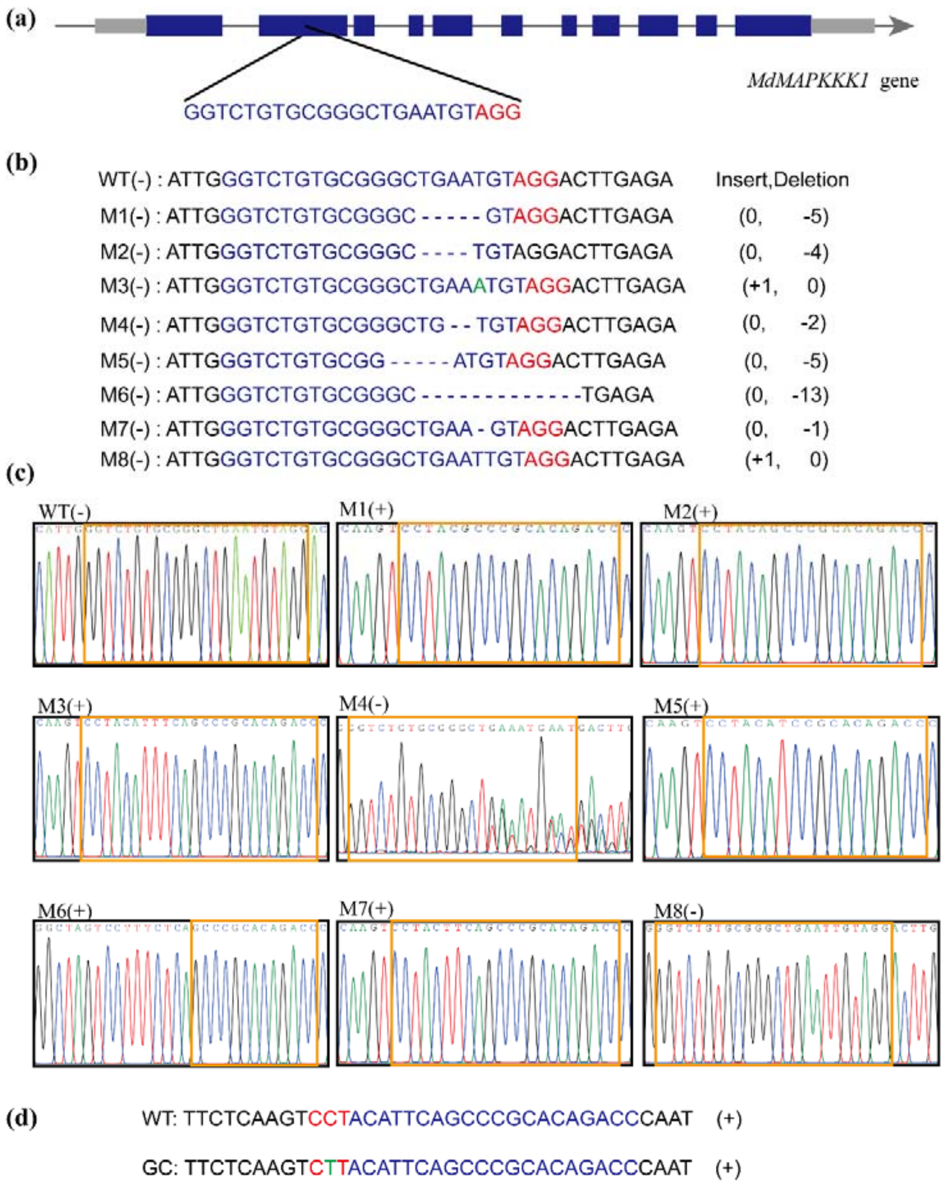

Figure S2. CRISPR/Cas9 mediated knockout of the *MdMAPKKK1* gene in apple callus. (a), Diagram of the *MdMAPKKK1* gene and targeting site for gene editing. Blue bars indicate exons. (b), The gene mutations detected in gene-edited calli. M1-M8, Different mutations in calli. (c), Sequencing chromatogram of partial genomic DNA corresponding to targeting sites of the *MdMAPKKK1* gene. (d), The sequence of site-directed mutagenesis of PAM site.

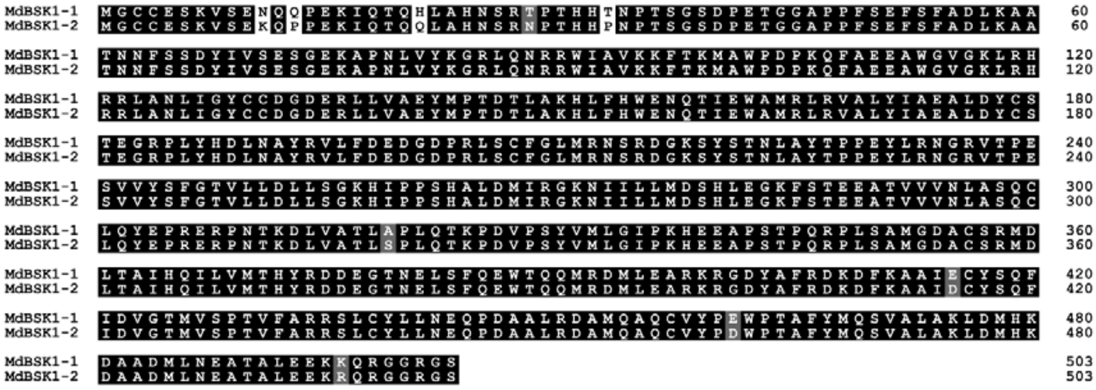

Figure S3. Alignment of MdBSK1-1 and MdBSK1-2. White letters shaded with black indicate identical amino acids and those with gray background indicate similar amino acids.

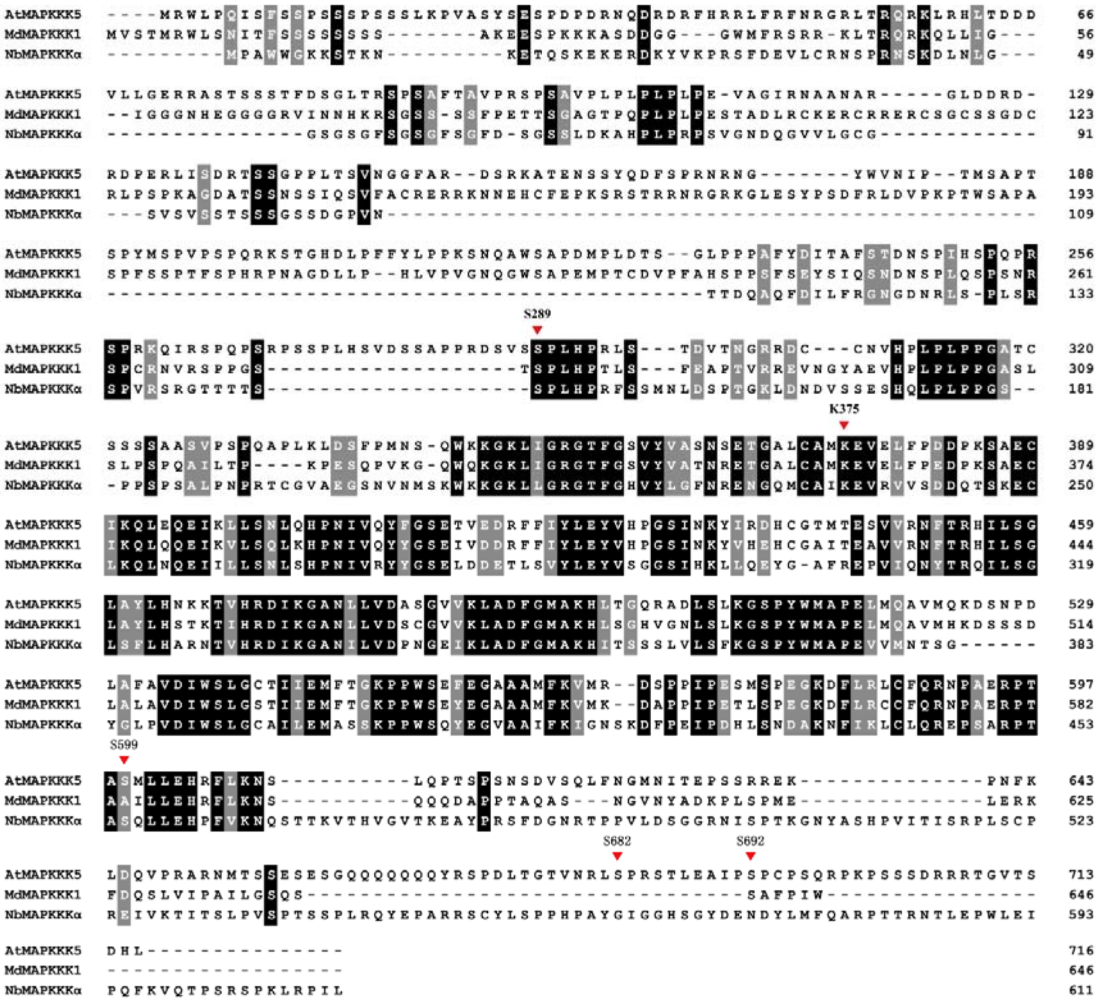

Figure S4. Multiple alignment of MAPKKK proteins. MAPKKK5, MdMAPKKK1, MAPKKKα were from Arabidopsis, apple (*Malus* × *domestica*), *Nicotiana benthamiana.* White letters shaded with black indicate identical amino acids and those with gray background indicate similar amino acids. Red arrows indicate the key amino acids of AtMAPKKK5. K375 is an ATP binding site and the other amino acids label with red arrows are phosphorylation sites.

Table S1. Genes used in the present study

Gene name	species	ID
MdMAPKKK1	*M. domestica*	MD02G1167500
MdMAPKKK2	*M. domestica*	MD04G1217700
MdMAPKKK3	*M. domestica*	MD12G1234900
MdMAPKKK4	*M. domestica*	MD12G1219600
MdMAPKKK5	*M. domestica*	MD11G1055100
MdMAPKKK6	*M. domestica*	MD03G1053500
MAPKKK5	*A. thaliana*	AT5G66850
ANP1	*A. thaliana*	AT1G09000
EDR1	*A. thaliana*	AT1G08720
AtMAPKKK3	*A. thaliana*	AT1G53570
MKD1	*A. thaliana*	AT5G11850
NbMAPKKKα	*N. benthamiana*	AAS78639
MdPBL27	*M. domestica*	MD13G1172400
MdPBL19	*M. domestica*	MD04G1053100
MdBSK1-1	*M. domestica*	MD05G1131400
MdBSK1-2	*M. domestica*	MD10G1134600
MdEF1-α	*M. domestica*	*MD04G1011000*

Table S2. The primers used in this study

Primer name	Primer sequence (5'→3')
**The primers for amplification of full-length cDNA**	
FL-MdMAPKKK1-F	ATGGTTTCCACCATGCGTTG
FL-MdMAPKKK1-R	TCACCAAATCGGGAATGCAC
FL-MdBSK1-1-F	ATGGGTTGCTGTGAATCCAA
FL-MdBSK1-1-R	TCATGATCCCCGGCCTCCTC
FL-MdBSK1-2-F	ATGGGTTGCTGTGAATCCAA
FL-MdBSK1-2-R	TCACGATCCCCGGCCTCCTC
FL-MdPBL19-F	ATGAAGTGTTTCTTTTATTT
FL-MdPBL19-R	CTACTTGGGTCTCCGCTTCG
FL-MdPBL27-F	ATGGGTGGGTGCTTTCCTTG
FL-MdPBL27-R	TCACTCATTTGTTCCGTCAA
**Quantitative real-time PCR primers**	
MdMAPKKK1-F	CTTAGCCTACCATCACCACA
MdMAPKKK1-R	CACATAACGCTCCAGTTTCT
MdBSK1-1-F	TTTGCCCGACGCAGTTTATG
MdBSK1-1-R	GCCTCCTCGCTGCTTCTTTT
MdBSK1-2-F	TACTGAGGGCCGTCCGTTAT
MdBSK1-2-R	GCGTGTAAGCGAGATTTGTG
MdEF1-α-F	CAAGGCAAGGTACGAGGAA
MdEF1-α-R	GAAGTGGAAGACGGAGGG
MdMAPKKK4-F	CTGAGGCAGTAATACAAACG
MdMAPKKK4-R	TTCATAGACTTAGCACCAGA
MdMAPKKK6-F	AGGCAGACAAGGCTGGCTAT
MdMAPKKK6-R	ACCTCCATCCCAAGCAACTC
MdMAPKKK2-F	GAGTCCTTCGTCGTCTTCGC
MdMAPKKK2-R	CGCCGCCTCAACCTTATCTC
MdMAPKKK3-F	AGGGAGAATCATTTCCAACA
MdMAPKKK3-R	CATCCTCATACCACCATCAA
MdMAPKKK5-F	ACCTCCTAAGCACCCTCAAC
MdMAPKKK5-R	TTATGGCTCGGATGTCTACC
**Primers for inducible overexpression of MdMAPKKK1**	
pER8-MdMAPKKK1-F	CCGCTCGAGATGGTTTCCACCATGCGTTG
pER8-MdMAPKKK1-R	GGACTAGTTCACCAAATCGGGAATGCAC
**Primers for construction of Crispr/CAS9 vector**	
MdU6-pHDE-F	GTCAAACACTGATAGTTTAAACCCGCAAGGAATTTAAGTT
MdMAPKKK1-T-MdU6-R	ACATTCAGCCCGCACAGACCAAGCATTACCTTCAAGTAAG
gRNA-MdMAPKKK1-T-R	GCTATTTCTAGCTCTAAAACACATTCAGCCCGCACAGACC
MdMAPKKK1-GC-F	AGTCtTACATTCAGCCCGCACAGACCCAATGC
MdMAPKKK1-GC-R	GGGCTGAATGTAaGACTTGAGAAAGGACTAGCCGGA
MAPKKK1-pro-F1	tttctcaagatcagaagtactTGTTTTCGTTAGCTGCACCT
MAPKKK1-pro-R1	tgtgattgtgatgtatctagaTCATGATCATCACAACCGCG
pRI101-MdMAPKKK1-GC-F	CACTGTTGATACATATGCCCATGGTTTCCACCATGCGTTG
pRI101-MdMAPKKK1-GC-R	GAGTTGTTGATTCAGAATTCTCACCAAATCGGGAATGCAC

**Primer for detection of mutation in target sequence**	
Crispr-MdMAPKKK1-649-F	TTCTTGCGTTGTTTGTTG
Crispr-MdMAPKKK1-649-R	CTGGGACTCTGGTTTAGG
**Quantitative real-time PCR primers**	
MdMAPKKK1-F	CTTAGCCTACCATCACCACA
MdMAPKKK1-R	CACATAACGCTCCAGTTTCT
MdBSK1-F	TTTGCCCGACGCAGTTTATG
MdBSK1-R	GCCTCCTCGCTGCTTCTTTT
MdEF1-α-F	CAAGGCAAGGTACGAGGAA
MdEF1-α-R	GAAGTGGAAGACGGAGGG
MdMAPKKK4-F	CTGAGGCAGTAATACAAACG
MdMAPKKK4-R	TTCATAGACTTAGCACCAGA
MdMAPKKK6-F	AGGCAGACAAGGCTGGCTAT
MdMAPKKK6-R	ACCTCCATCCCAAGCAACTC
MdMAPKKK2-F	GAGTCCTTCGTCGTCTTCGC
MdMAPKKK2-R	CGCCGCCTCAACCTTATCTC
MdMAPKKK3-F	AGGGAGAATCATTTCCAACA
MdMAPKKK3-R	CATCCTCATACCACCATCAA
MdMAPKKK5-F	ACCTCCTAAGCACCCTCAAC
MdMAPKKK5-R	TTATGGCTCGGATGTCTACC
**Primers for VIGS**	
VIGS-MdMAPKKK1-300-F	CGGAATTCAGAGTCCCAGCCAGTCAAAG
VIGS-MdMAPKKK1-300-R	CGGGATCCACATATTTGTTAATAGAACC
VIGS-MdBSK1-F	ATTCTGTGAGTAAGGTTACCAGGCCCCTAATCTTGTTTAC
VIGS-MdBSK1-R	AGACGCGTGAGCTCGGTACCAACACTTTCTGGAGTGACCC
**Primers for site-directed mutagenesis**	
MdMAPKKK1^{K360M}-F	TGTGCAATGATGGAAGTCGAGTTATTTCCTGAGGACCCAAAA
MdMAPKKK1^{K360M}-R	CTCGACTTCCATCATTGCACATAACGCTCCAGTTTCTCTATT
**Primers for Y2H and BiFC**	
MdMAPKKK1-BD-F	CGGAATTCATGGTTTCCACCATGCGTTG
MdMAPKKK1-BD-R	CGGAATTCATGGTTTCCACCATGCGTTG
MdBSK1-AD-F	ATATGGCCATGGAGGCCAGTATGGGTTGCTGTGAATCCAA
MdBSK1-AD-R	ATCTGCAGCTCGAGCTCGATTCATGATCCCCGGCCTCCTC
FL-MdMAPKKK1^{K360M}-NLUC-F	CGGGATCCATGGTTTCCACCATGCGTTG
FL-MdMAPKKK1^{K360M}-NLUC-R	GCGTCGACTCACCAAATCGGGAATGCAC
MdBSK1-CLUC-F	GCGTCCCGGGGCGGTACCCGATGGGTTGCTGTGAATCCAA
MdBSK1-CLUC-R	TACGAACGAAAGCTCTGCAGTCATGATCCCCGGCCTCCTC
